# Moral judgement development during medical student clinical training

**DOI:** 10.1186/s12909-021-02572-4

**Published:** 2021-03-02

**Authors:** Jenny McDonald, Jane Graves, Neeshaan Abrahams, Ryan Thorneycroft, Iman Hegazi

**Affiliations:** 1 School of Medicine, Western Sydney University, Penrith, NSW Australia; 2 School of Sociology, Western Sydney University, Penrith, NSW Australia

**Keywords:** Medical students, Professionalism, Morals, Medical education

## Abstract

**Background:**

Whereas experience and cognitive maturity drives moral judgement development in most young adults, medical students show slowing, regression, or segmentation in moral development during their clinical years of training. The aim of this study was to explore the moral development of medical students during clinical training.

**Methods:**

A cross-sectional sample of medical students from three clinical years of training were interviewed in groups or individually at an Australian medical school in 2018. Thematic analysis identified three themes which were then mapped against the stages and dimensions of Self-authorship Theory.

**Results:**

Thirty five medical students from years 3–5 participated in 11 interviews and 6 focus groups. Students shared the impacts of their clinical experiences as they identified with their seniors and increasingly understood the clinical context. Their accounts revealed themes of early confusion followed by defensiveness characterised by desensitization and justification. As students approached graduation, some were planning how they would make moral choices in their future practice. These themes were mapped to the stages of self-authorship: External Formulas, Crossroads and Self-authorship.

**Conclusions:**

Medical students recognise, reconcile and understand moral decisions within clinical settings to successfully reach or approach self-authorship. Curriculum and support during clinical training should match and support this progress.

## Background

Modern medical practice requires the synthesis of knowledge, skills and wisdom to ensure optimal care matches patient need, context and available resources. This requires not only access to the latest literature and clinical guidelines, but sound moral judgement [1]. In contradistinction to ethical decisions based on agreed principles, moral judgement in clinical practice is based on personal beliefs about what is right and wrong for the patient combined with the motivation to do the right thing [2]. Moral judgement influences professional practice and shapes identity development [3]. Medical students need to graduate with the confidence to make moral decisions [4–6] about patient care and their professional behaviour while abiding by the ethical guidelines for practice. For medical students, who are usually in early adulthood, neuro-maturation, social context, relationships and experience drive moral judgement development [7]. The formal curriculum and the ‘hidden curriculum’ in medical courses, embedded in learning experiences and role modelling within clinical settings, both have a role to play [8].

Several factors have been proposed to cause the slowing or regression in the moral development of medical students and to explain why medical education appears to be the exception to the rule that tertiary education fosters moral development [9] These factors include unsupported exposure to ethical dilemmas, negative clinician role modelling in the hospital setting [4, 10–12]; inequities in patient services and care; being placed in a position to act unethically [13]; the powerlessness of medical students [14, 15]; and the socializing experience of medical school [16]. Feudtner et al. [13] found a correlation between witnessing unethical behaviour, acting unethically and the reported sense by medical students that ethical principles and their moral judgement had been violated and compromised. The inability to act upon moral judgements causes moral distress [17], and this emotional response may also be a factor in the disruption of moral development. Medical students are particularly vulnerable to moral distress because of their low place in the healthcare system hierarchy. They are required to accept the right answer in a clinical setting rather than to use critical reasoning to solve problems [9] or their moral judgement.

To address challenges to moral judgement in medical students, formal teaching in ethics has been shown to enhance moral development [18] and reduce moral distress [11]. However, this may lead to conflict between the virtues taught in the formal curriculum and what the students actually experience through the hidden curriculum. This in turn may lead to moral relativism and cynicism among students as they progress through medical school [19]. Students who are able to reflect effectively may be better place to tolerate this conflict, as a correlation has been shown between lower reflection ability and moral judgement regression in final year medical students [20]. Timing and understanding of moral judgement development is critical for interventions to be effective.

Piaget [21] and Kohlberg [22] laid the groundwork for the current understanding of moral judgement development. Quantitative longitudinal studies, using standardised tests of moral judgement based on Kohlberg’s theory, have explored moral development during medical training. Lind’s group [23] compared medical students to other tertiary students. Medical students showed a decline in their moral judgement competence, whereas other tertiary students showed growth (as would be expected in early adulthood). Other studies [9, 20, 24, 25] have reported regression in moral judgement in medical students, coinciding with exposure to clinical settings. Medical students showed a shift towards a less mature approach to moral judgement, with precedence given to pleasing others rather than social justice imperatives. In other words, there was a retrograde shift towards what Kohlberg described as conventional rather than post-conventional level of moral reasoning [22]. In other studies, there has been evidence of moral segmentation [26, 27]. Medical students applied lower level of moral reasoning to a moral dilemma within a clinical setting compared to a non-clinical setting as their training progressed.

To explain the complexity in moral judgement development, Kohlberg’s theory has been modified and expanded to explain differences in moral reasoning according to gender [28] and social context. Gilligan [29], in her Theory of Moral Orientations, argued that females are more concerned with care and social relationships compared to males who prioritise justice during moral judgements. In the more recent Moral Foundations Theory [30], there has been a shift towards the exploration of dimensions of moral judgement beyond justice and care to include group loyalty and respect for authority. This allows an understanding of similarities and differences in individual and societal moral concerns.

This dimensional conception of moral judgement is in keeping with modern theories of development, such as Dynamic Systems Theory (DST) [31]. It also helps to explain the early research findings showing disruption and variations in moral judgement development. DST states there is more variation in the development of individuals than in groups. This means that the average group progress and developmental stages do not explain individual developmental trajectories. An individual’s development arises in an iterative fashion, but is not always linear and is multi-dimensional; some dimensions may progress faster than others. Positive, negative or absent feedback loops influence these developmental dimensions. This feedback process leads to development with more stable patterns appearing over time that restrict less mature patterns.

For our study, we have chosen Self-authorship theory [32] to guide our analysis as it provides a developmental and dimensional framework for moral judgement development as proposed in DST. Self-authorship theory, first proposed by Kegan [33] and expanded by Baxter Magolda in her studies of young adults [32], conceptualises the development of self-authorship as a constructive-developmental process whereby young adults shift from relying on external authorities to internalised values as the basis for their moral judgements [7]. In Self-authorship theory, there are three dimensions: epistemological, intrapersonal and interpersonal and three stages: the ‘external formulas’ stage (where knowledge is understood initially as absolute, giving way to an understanding of knowledge as contextual); the ‘crossroads’ stage (a period of uncertainty or disequilibrium); and the self-authorship stage (reached by some in the third or fourth decade, where an individual has an established value or belief system and sense of identity). These stages and dimensions are summarised in Table 2.

In this study, we explore medical student moral judgement development in the clinical setting through the lens of Baxter Magolda’s self-authorship theory [7]. This study builds on the quantitative studies of moral development in medical students and the studies of medical student experiences in clinical settings more generally. Understanding the development of moral judgement development in medical students provides a timeline for curriculum design that appropriately educates supports and challenges students.

## Methods

### Aim

The aim of this study was to explore the moral judgment development of medical students during their clinical training.

### Setting

At ***blinded***School of Medicine, the Personal and Professional Development curriculum includes teaching in patient centred care, ethics in medicine and research, advocacy, critical thinking, personal and professional identity and well-being [34]. This formal teaching occurs throughout the course but is concentrated in the first 2 years. For their final 3 years, students learn through immersion in clinical settings in busy outer metropolitan and rural hospitals.

### Study design

A qualitative approach was adopted given the sensitive and subjective nature of moral judgement. Participants were offered the opportunity to speak with a researcher as individuals or as part of a focus group. We were interested in changes in moral judgement so participants were recruited from the 3 years of clinical training. This study has been conducted according to relevant guidelines and regulations. We have followed the qualitative research reporting guidelines recommended by O’Brien et al. [35].

### Participant recruitment

Students enrolled in ***blinded*** School of Medicine were invited to participate through the student email system and announcements on the student learning management system. All students, enrolled in the clinical years of the program (Years 3–5) who volunteered to participate were recruited to the study. Between July and December 2018, the researcher RT conducted 11 interviews and 6 focus groups with 35 medical students (including 18 students from 3rd year, 11 students from 4th year and 6 students from 5th year) drawn from a school cohort of 600 students. Informed consent was provided prior to interview. There were 15 female and 20 male participants with approximately equal representation for each year. The participants were offered an interview or inclusion in a focus group. The focus groups ranged in size from four to six participants.

### Data collection and analysis

A research assistant, RT, who was not involved in teaching our participants, conducted semi-structured interviews and focus group discussions lasting approximately 60 min. All interviews were audio recorded and transcribed verbatim. Questions with prompting allowed participants to describe their first impressions, experiences and responses during clinical placements to elicit spontaneous accounts of moral judgements and responses. The students were also asked directly about the role of moral judgement in clinical practice; their perceptions of how their moral judgement was changing and the role of the medical school in supporting moral judgement. The approach to interviews was adapted following team review of the student responses in the first two sessions. See Table 1.

**Table 1:** List of interview questions

1. Can you tell me a little about yourself?	
2. How much clinical placement or clinical experience have you had so far?	
3. Can you talk a little about your experiences on clinical placement?	
4. Can you describe the hospital culture and how it compares to other places you have worked or studied?	
*Statement: Moral judgment is defined as “the process by which an individual determines what’s right and wrong, good and bad”.*	
5. How important do you think moral judgement is in a clinical practice?	
6. Have your ideas about what is right and wrong changed?	

De-identified data was thematically analysed, through an iterative and reflexive approach as described by Braun and Clarke [36]. The researchers were blinded to participant gender but not year of study for the analysis. JG and NA independently applied initial descriptive codes to participants’ responses in the interviews and focus group discussions. They discussed and refined these codes during this process. JM reread the transcripts, reviewed and consolidated these initial codes into broader topics. Data saturation was determined when no new topics or insights were identified in the data [37]. At this point, no further participants were recruited. We used QSR International Pty Ltd. (2020) NVivo (released in March 2020) to manage the analysis.

The final themes were discussed, reviewed and refined until consensus was reached that they sufficiently explained the development of the medical students’ moral judgements. These were mapped to the dimensions and stages of Self-Authorship Theory. This was undertaken by creating a grid with Baxter Magolda’s criteria of Self-authorship [32] on one axis and our themes against the other. The criteria for Baxter Magolda’s self-authorship stages and dimensions [32] are summarised in Table 2.

**Table 2:** Criteria for Dimensions and Stages of Self-Authorship

Dimension	External Formulas	Crossroads	Self-authorship
Epistemological	Knowledge from authorities is accepted or partially accepted without evaluation	Evolving awareness of the uncertainty created by multiple perspectives and recognition of the need to accept responsibility for beliefs	Knowledge is contextual. An internal belief system allows the construction, evaluation and interpretation of evidence to form judgements
Intrapersonal	Identity derived from the definitions and approval of others	Emerging tension between internal and external values and beliefs prompting self-exploration	Consolidation of personal values and identity to allow interpretation of evidence within context
Interpersonal	Relationships are a source of identity and affirmation	Evolving recognition of how dependent relationships constrain growth. Emerging identity creates struggle for independence	Engagement in interdependent relationships without the need for approval and with the ability to recognise and accept the perspectives of others

(Adapted from Baxter Magolda M, King PM. Learning partnerships: Theory and models of practice to educate for self-authorship: Stylus Publishing, LLC.; 2004. with permission of the publisher. Copyright© 2004, Stylus Publishing, LLC).

The team engaged in reflexivity to consider the influence of personal bias in the analysis of the data. The researchers JM, JG and NA have taught components of the personal and professional development course to the participants and JM has worked in one of the hospital settings. The researchers sought to distinguish between the taught curriculum and the participant responses, and to set aside, where possible, any personal knowledge of clinical culture and practice in the analysis of the data.

Once established, the themes were mapped against the stages and dimensions of self-authorship theory (epistemological, intrapersonal and interpersonal).

## Results

Three main themes were identified during the focus group discussions and interviews. Some themes were more prominent in the early years, representing a time of moral judgement questioning and emotional responses to early clinical experiences. Other themes became more prominent in the later years of training as students adapted to the clinical environment, developed confidence, and began planning for how they would approach moral dilemmas in their future clinical practice. The three themes that emerged were confusion, defensiveness (desensitization and justification) and planning for the future.

### Confusion

The students described wide ranging new experiences both favourable and unfavourable related to patient care, doctor-patient communication, and team dynamics. In third year, students described confusion when moral decision-making within clinical practice did not match their expectations:

“It probably drops the most in the first clinic that you have because of the difference between the idealism you're taught in school and the actual real-life experience”. (P22, Year 3, female)

This meant they felt powerless to respond:

“So I guess idealistically you’d want to say you’d step in and do what you think is morally correct, but I didn’t in that situation. I just didn’t really ... I was kind of in shock”. (P31, Year 3, male).

Medical students described being highly motivated to please their seniors and being somewhat in awe of their knowledge and competence. This means that initially they doubted their own moral competence:

“I think when I saw scenarios that were maybe slightly morally questionable; I was still understanding what was normal for the hospital”. (P18, Year 4, female).

“So, when you see your superior in that position behaving a certain way it just sort of comes to you that you need to develop that attitude and mindset”. (P12, Year 4, female).

“… you have people telling you in higher positions, telling you to do something, you just assume that it’s right and it’s the right decision to make at the time. That’s just what’s expected”. (P28, Year 3, male).

This respect for the senior clinicians meant conforming to rules to please others. This corresponds to the External Formulas phase of self-authorship.

However, conforming to hospital culture conflicted with their moral judgement:

“We can’t often speak up for when we see bad behaviour, but we do make our own judgements and we talk about it amongst ourselves as well”. (P14, Year 4, male).

### Defensive

This conflict between the clinical practice and the participants’ moral judgements elicited two defensive responses.

#### Desensitization

The first of these responses was desensitization with moral detachment:

“I remember the first time I was around a doctor telling a patient’s family that the patient was going to die, I got quite upset. And it happened again a few months later, and I felt nothing … I have kind of fallen into the trap of disengaging emotionally”. (P7, Year 3, female).

I feel like that’s the normal process of becoming a doctor. As long as you’re not completely insensitive but, yeah, I’m more open to rejecting people now. In the past, I wouldn’t reject anyone like if they want to talk. Now, I would if it’s necessary. (P1, Year 3, male).

#### Justification

The second defense was justification. Participants justified their desensitisation to distressing situations or problematic behaviours:

I think eventually your sense of being actively involved and emotionally affected by the patient’s plight will decrease, and I think that’s just natural. (P17, Year 4, male).

Participants also justified their questionable moral choices by expediency. Their need to survive medical school outweighed the priority for moral actions in terms of speaking out:

“… while we learn about you can stand up to your superiors, and it’s really good, but in reality, that’s always in the back of our mind, because our ultimate goal is to just try and survive through medical school”. (P13, Year 4, female).

“In theory, they talk about being able to stand up for yourself … in practice, it’s so difficult and it is because like, because it’s so hierarchical …” (P5, Year 5, female).

This justification subverted moral judgement. Choices and actions were determined by the best interests of the student or the rules of the prevailing culture. External Formulas were more important than moral judgement.

Participants also justified the desensitisation of their seniors who needed to cope with what they were learning, by pitching the blame to an unforgiving system with limited resources:

“I don’t truly believe all these people became, that they were born that way. I think they became jaded somehow”. (P21, Year 3, male).

Yet some students were aware of and distressed by their increasing insensitivity. They felt there was a disconnection between their ideals, what they were seeing and increasingly accepting. This disequilibrium represents the Crossroads of Self-authorship:

“And you see it in senior doctors. They kind of do seem to, a lot of the time, see patients as a problem to solve, rather than just a person”. (P7, Year 3 female).

In the fifth-year focus group discussion, the students argued that the responsibility for their moral judgement lapses lay with their medical educators – another defensive justification. This response shifted responsibility for moral action on others, almost abdicating personal responsibility. These responses contained potential solutions for the moral distress they had experienced:

“I think while we were transitioning from preclinical to purely clinical, it was very hard to adapt to a clinical learning, and I felt quite lost like what to do, how long do I stay here, how do I learn? ... I think a little bit more teaching during that period and support and debriefing sessions formally by the school would have been good during that”. (P2, Year 5, female).

### Planning for the future

In their final years, students discussed how they would use what they had learned from experience in their future practice. They were cautiously confident they could do better in terms of relationships with juniors:

“I often think about, as an intern, like it’ll be the first time we have juniors underneath us and I often think about like, how can I change the experience that I’ve had for my future medical students?” (P4, Year 5, female).

And moral practice:

“We’re learning how not to be doctors for the most part. You get a lot of good doctors but the thing that definitely stands out to me is, wow, I need to make sure I never do that to anyone ever again because that’s awful”. (P34, Year 5, male).

This represents an early shift towards self-authorship with consolidation of personal values and professional identity.

**Fig. 1:**
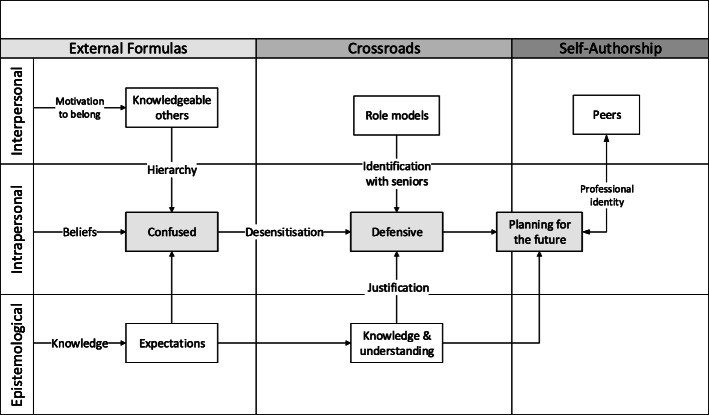
The resulting themes mapped against the stages and dimensions of self-authorship_B&W

The three themes are mapped against the three stages and dimensions of self-authorship in Fig. 1.

## Discussion

In our study, the students’ moral judgements varied widely and depended on context. Across the years, there was evidence of developmental growth in moral judgement. Early confusion and defensiveness made way for greater confidence in moral judgement as students consolidated their professional identities in the backdrop of a complex environment. As dynamic systems theory [31] predicts the path was not linear for groups or individuals.

Modern medical curricula adopt an immersive style of teaching drawing on socio-cultural learning theories where students learn through joining a community of learners through participation in work [38]. This approach to preparing graduates for practice is not only theoretically sound but has a strong evidence base [39]. Therefore, it is no wonder that in their early transition to the clinical setting students show eagerness to impress their seniors, to learn from them and to be accepted into the culture. Hafferty and Franks [40] argue that ‘medical students suffer from professional insecurity and fear of failure, and that they generalize this perceived incompetence as ethical incompetence as well’. Students, hence, see undesirable practices and treat them as representative of what is acceptable in the face of what they have learned and previously believed. This allows the ‘hidden curriculum’ to predominate over personal morality.

In our study, the third-year students quickly recognised the hierarchical nature of healthcare settings and described feeling confused: powerless to protest, unknowledgeable and willing to trust their superiors. This led to an uneasy adoption of an External Formulas approach to moral judgement; students yielded their knowledge and beliefs to others because they are uncertain about their own values and social identity [32].

The modern clinical setting is a complex and dynamic environment driven by relationships and system processes that are interspersed by critical events. The students’ experiences provided a sharp contrast between the reality of clinical practice and the explicitly taught professionalism ideals. The incongruence between what the students experience and their ideals led to distress-provoked early confusion followed by defensiveness. Students become caught in what Baxter Magolda [32] refers to as the ‘crossroads space’, characterised by tension and uncertainty as young adults attempt to reconcile new experiences with an emerging identity and belief system. This disequilibrium is described as a necessary stage and driver towards self-authorship [41]. Identity development according to DST is characterised by cycles of commitment (identity) and exploration for solutions [42].

Students became defensive during this period of uncertainty resorting to two main solutions to resolve the conflict: desensitisation and justification. Many described becoming insensitive and found this both protective and alarming. Others sought to justify their superiors’ behaviours reflecting a shift towards greater identification with their seniors and acceptance of the complex nature and competing priorities in health care. Some students attempted to justify their behaviours that did not conform to their ideals. These students just wanted to ‘get through’ their training, complete their logbooks and ‘survive’. Many students also felt angry that they had been let down by their training, particularly the perceived inadequacy of the support they had received. These students advocated strongly for more opportunities to debrief about their experiences on the wards and for teaching in assertiveness and ethical reasoning.

In their final year, students expressed determination to create cultural change within the clinical setting and the medical curriculum as they began to plan for their clinical practice. This represents an early shift towards accepting responsibility for moral judgements and the final stage in the self-authorship process where young adults take responsibility for their values, respect the perspectives of others, while no longer being dependent on the approval of others for their own beliefs and behaviours. Segmentation of moral competence, described in earlier studies can be explained by DST as a fracturing of moral competence in a new and bewildering context. Students resolve this inconsistency in their moral judgement as they consolidate their moral judgement through self-authorship. This development is likely to be unique to the individual, dynamic and contextual. The implications of this research are that students are at risk of being underprepared and under supported for their exposure to the clinical setting. To address this, medical educators need to prepare students for the reality of the complexity of medical workplaces [39]. Once immersed in a clinical setting, Sandars and Jackson [43] recommend structuring support for medical student through Baxter Magolda’s learning partnership model [32] which takes into account the student’s self-authorship stage. The knowledge that students need to attain to make sound moral judgements in clinical practice is complex. This knowledge needs to be presented in an iterative fashion. Students need the opportunity to reflect and discuss ‘disorienting’ experiences that challenge ideals and learned theoretical concepts. Many of the students recounted the focus groups and interviews were one of the few opportunities given to debrief their experiences in any meaningful way. Students should be challenged to consider how they might do things differently as they reach self-authorship and develop confidence in their moral judgement.

It is also the responsibility of medical educators to ensure that learning environments for our students are safe and stimulating. Role models, positive and negative, have a powerful influence on student values, motivations and behaviours [44]. Toxic health care environments are neither beneficial to patient care nor the well-being of medical students [45]. Our medical students and junior medical officers need the confidence, skills and opportunity to speak up when they are witness or victim to unprofessional behaviours [46]. This requires that students have the confidence and means to act on their moral judgements.

This study was cross-sectional with representation of three years of a medical cohort. This allowed comparison of perspectives of students at different stages. A longitudinal qualitative study may have allowed exploration of individual factors influencing student journeys. Our sample of students was drawn from a single university and participants self-selected. Their experiences, while reflective of what has been reported in the literature, may not be representative of their respective year cohorts or of medical students from other universities. Future research should explore the development in moral judgement in young graduates as they become more senior, accept more responsibilities and progress through postgraduate training. It will also be important to test intervention effectiveness on medical student experience and moral development.

## Conclusion

This study explored how the moral judgement development of medical student during clinical training. Our findings reflect and build upon the findings in earlier studies that have found student distress on entering a clinical environment. The students described how their eagerness to join and conform to the medical community culture influenced their early responses to clinical experiences. Initial confusion and distress provoked a lack of confidence and questioning of their moral judgement. By final year, most students had shifted towards self-authorship by making sense of their experiences and the clinical environment, reconciling what they had learned with their ideals and forging plans for their future practice. Medical educators should anticipate this developmental process, providing preparation and support during transitions, opportunities for reflection during the Crossroads period of disequilibrium and provide challenges for students as they develop self-authorship maturity to express and exercise their moral judgement.

## Data Availability

The datasets generated and/or analysed during the current study are not publicly available due to the personal nature of the topic and the potential for participant re-identification. Aggregate de-identified data is available on reasonable request.
